# Differential Expression of Inflammation-Related Genes in Children with Down Syndrome

**DOI:** 10.1155/2016/6985903

**Published:** 2016-05-11

**Authors:** Cláudia Regina Santos Silva, Joice Matos Biselli-Périco, Bruna Lancia Zampieri, Wilson Araujo Silva, Jorge Estefano Santana de Souza, Matheus Carvalho Bürger, Eny Maria Goloni-Bertollo, Érika Cristina Pavarino

**Affiliations:** ^1^Unit of Research in Genetics and Molecular Biology (UPGEM), Department of Molecular Biology, Medical School of São José do Rio Preto (FAMERP), 15090-000 São José do Rio Preto, SP, Brazil; ^2^Department of Genetics, Ribeirão Preto Medical School, University of São Paulo, Ribeirão Preto, SP, Brazil; ^3^Center for Medical Genomics at HCFMRP/USP, Ribeirão Preto, SP, Brazil; ^4^Regional Blood of Ribeirão Preto at HCFMRP/USP, Ribeirão Preto, SP, Brazil; ^5^Institute of Bioinformatics and Biotechnology (2bio), Ribeirão Preto, SP, Brazil; ^6^Metrópole Digital Institute (IMD), UFRN, Natal, RN, Brazil

## Abstract

*Objective*. The aim of the study was to investigate the expression patterns of a specific set of genes involved in the inflammation process in children with Down Syndrome (DS) and children without the syndrome (control group) to identify differences that may be related to the immune abnormalities observed in DS individuals.* Method*. RNA samples were obtained from peripheral blood, and gene expression was quantified using the TaqMan® Array Plate Human Inflammation Kit, which facilitated the investigation into 92 inflammation-related genes and four reference genes using real-time polymerase chain reaction (qPCR).* Results*. Twenty genes showed differential expression in children with DS; 12 were overexpressed (*PLA2G2D*,* CACNA1D*,* ALOX12*,* VCAM1*,* ICAM1*,* PLCD1*,* ADRB1*,* HTR3A*,* PDE4C*,* CASP1*,* PLA2G5,* and* PLCB4*), and eight were underexpressed (*LTA4H*,* BDKRB1*,* ADRB2*,* CD40LG*,* ITGAM*,* TNFRSF1B*,* ITGB1,* and* TBXAS1*). After statistically correcting for the false discovery rate, only the genes* BDKRB1* and* LTA4H* showed differential expression, and both were underexpressed within the DS group.* Conclusion*. DS children showed differential expression of inflammation-related genes that were not located on chromosome 21 compared with children without DS. The* BDKRB1* and* LTA4H* genes may differentiate the case and control groups based on the inflammatory response, which plays an important role in DS pathogenesis.

## 1. Introduction

Down Syndrome (DS), which is also referred to as trisomy of chromosome 21 (HSA21), is the most common human aneuploidy (1/660 births) [1], and it is characterized by intellectual deficiency [2], a variety of dysmorphic physical characteristics [3, 4] and a variable spectrum of clinical manifestations [5, 6], including immunodeficiency [7].

Infections, especially in the respiratory tract [8, 9] and autoimmune diseases, including diabetes mellitus [10], primary hypothyroidism [11], and celiac disease [12], occur more frequently in individuals with DS than in individuals without the syndrome [13]. In addition, infectious diseases are a significant cause of hospitalization and mortality in individuals with DS [14, 15].

The aetiology underlying the immunological deficiency has not been fully described. However, changes in the immune system, such as functional and morphological thymus abnormalities [16, 17]; changes in T-cell differentiation, maturation, and activation; and lymphopenia [18–20], suggest a prematurely senescent phenotype in the immune systems of individuals with DS [21]. Such changes may contribute to increased susceptibility to infections and inflammatory processes.

Gene expression studies in individuals with DS have reported differential expression of genes involved in immunological processes, which may explain the immunodeficiency observed in these individuals [22–27]. In fact, the imbalance due to the extra copies of genes on chromosome 21 and the effects of these gene products of other disomic genes or on specific proteins activities may explain how trisomy 21 results in the DS phenotype [3, 28].

In this study, we analysed the expression patterns of 92 genes involved in inflammatory processes in children with DS and children without the syndrome (the control group) to identify differences that may be related to the immunological abnormalities observed in individuals with DS.

## 2. Materials and Methods

### 2.1. Samples and Groups

Six individuals with DS (four males and two females) with a mean age of 3.2 years (ranging from 2.1 to 6.6 years) were referred to the study by the Genetics Outpatient Service of the Hospital de Base (HB), São José do Rio Preto, SP, Brazil. Of these children, five had free trisomy of HSA21, and one had mosaicism (90% of cells were trisomic). The control group consisted of six children without DS (one male and five females) with a mean age of 5.9 years (ranging from 3.9 to 6.5 years of age) who were undergoing routine care at the Outpatient Pediatric Clinic of the HB. Inclusion criteria were children aged between two and six years for both groups. In the control group, patients were included if they had absence of diseases associated with clinical manifestations of DS. Exclusion criteria for both groups were the presence of clinical signs that suggest acute infection, including cold symptoms, cough, fever, and/or antibiotic use, up to ten days prior to the data collection date, and the absence of chronic infections (bronchitis, asthma, and recurring pneumonias). We performed a C reactive protein analysis using serum samples and a Cobas C 501 Analyzer (Roche) to confirm the absence of infections. All samples included within the study were negative.

### 2.2. Total RNA Isolation and Quantitative Real-Time PCR (qPCR)

Peripheral blood samples (3 mL) were collected into a Tempus Blood RNA tube (Applied Biosystems®) containing 6 mL of RNA Stabilization Solution. Total RNA samples were isolated using the Tempus Spin RNA Isolation Kit (Ambion®). The RNA samples were quantified; their purities (*A*260/280 nm) were measured using spectrophotometry (Picodrop*™* 200, Thermo Scientific), and only samples with intact 18S and 28S fragments were used throughout the gene expression analyses.

Complementary DNA (cDNA) was synthesized using the High-Capacity cDNA Reverse Transcription kit (Applied Biosystems, Carlsbad, California, USA) in accordance with the manufacturer's instructions. The reverse transcription reaction products (12.5 ng) were analysed in duplicate using the TaqMan Array Human Inflammation 96-well Plate (Applied Biosystems, Carlsbad, California, USA) (catalogue number 4414074) in accordance with the manufacturer's protocol. The 96-plex gene card included 92 genes related to inflammation and four reference genes (Table 1). The reaction conditions were as follows: 95°C for 10 minutes followed by 40 cycles at 95°C for 15 seconds and 60°C for 1 minute in a StepOne Plus thermocycler (Applied Biosystems, Carlsbad, California, USA). Raw values throughout the quantification cycle (Cq) were generated using the software StepOne version 2.3 (Applied Biosystems) after we manually adjusted the threshold for each gene analysed.

### 2.3. Statistical Analysis

Gender and age distributions between groups were compared using Fisher's exact and Mann-Whitney tests, respectively, with the program GraphPad Prism version 5.01. *p* values less than 0.05 were considered significant.

Gene expression analyses were performed using the R statistical language [29] and free packages available from the Bioconductor repository [30]. The package HTqPCR [31] was used to preprocess the data and as an interface with other packages using the PCR data as input. A Cq value of 37 was used as the threshold for detectable gene expression because the qPCR data were normally distributed up to this value. The gene expression data were normalized using the quantile normalization method [32, 33]. A normal distribution of data was generated using the mean and standard deviation of the Cq values. Individual Cq values outside the interval set by “quantile” were considered “unreliable” and removed from the analysis.

The criterion for a valid statistical analysis of a given gene was the expression in at least 60% of the biological samples within each group. Differentially expressed genes were identified using linear models for microarray data (LIMMA) [34]. To reduce the risk of false positives, *p* values were adjusted for multiple testing using the Benjamini-Hochberg False Discovery Rate method [35] with *α* = 0.05. Gene's expression within the case group was expressed relatively to the control group expression (relative quantification, RQ).

### 2.4. Functional Analyses of Differentially Expressed Genes

The most highly represented biological processes from the differentially expressed genes in children with DS were identified using the software “The Database for Annotation, Visualization, and Integrated Discovery” (DAVID) version 6.7 [36, 37] and the Kyoto Encyclopedia of Genes and Genomes (KEGG) database. The significant results (*p* < 0.05) after correction for multiple tests are indicated [35].

## 3. Results

The ages (*p* = 0.093) and genders (*p* = 0.080) of the groups did not differ significantly, which indicates homogeneity between the groups.

### 3.1. Gene Expression Analyses

Of the 92 inflammation genes tested, 20 genes were differentially expressed between the children with DS and control children (*p* < 0.05) before correcting for multiple tests. Twelve genes showed increased expression (*PLA2G2D*,* CACNA1D*,* ALOX12*,* VCAM1*,* ICAM1*,* PLCD1*,* ADRB1*,* HTR3A*,* PDE4C*,* CASP1*,* PLA2G5,* and* PLCB4*), and eight genes showed decreased expression in children with DS (*LTA4H*,* BDKRB1*,* ADRB2*,* CD40LG*,* ITGAM*,* TNFRSF1B*,* ITGB1,* and* TBXAS1*) (Table 2). After a statistical adjustment for multiple tests, only two genes showed significantly different expression levels, Bradykinin receptor B1 (*BDKRB1*) and Leukotriene A4 hydrolase (*LTA4H*); the significance value was *p* = 0.02 for both genes, and both genes showed reduced expression in individuals with DS.

### 3.2. Functional Analyses of Differentially Expressed Genes

We analysed the metabolic pathways of 20 differentially expressed genes in children with DS. The analysis using the KEGG database classified the genes into three pathways. The calcium signalling pathway ranked first and included six genes. The two other pathways, the cellular adhesion molecule (CAM) and arachidonic acid metabolism pathways, included five genes (Table 3). According to this tool, the* BDKRB1* and* LTA4H* genes that showed statistically significant differential expression after correction for multiple tests are involved in calcium signalling pathways, as well as in the arachidonic acid metabolism.

## 4. Discussion

Of the 20 differentially expressed genes in children with DS in this study, 12 showed increased expression (*PLA2G2D*,* CACNA1D*,* ALOX12*,* VCAM1*,* ICAM1*,* PLCD1*,* ADRB1*,* HTR3A*,* PDE4C*,* CASP1*,* PLA2G5* and* PLCB4*), and eight showed decreased expression (*LTA4H*,* BDKRB1*,* ADRB2*,* CD40LG*,* ITGAM*,* TNFRSF1B*,* ITGB1* and* TBXAS1*). None of the genes are located on chromosome 21 (i.e., they have two copies in the individuals studied), which corroborates previous findings that trisomy 21 produces a global change in gene expression throughout the genome, not only for genes on chromosome 21 [4, 38]. The magnitude of these changes, whether large or small, is likely to be associated with the type of tissue analysed, and the different sensitivities of the various techniques used to analyse gene expression (SAGE, microarray, and qPCR) [39].

Functional analyses of the differentially expressed genes showed that genes* BDKRB1* and* LTA4H*, which showed significantly lower expression in children with DS even after a statistical adjustment for multiple tests, are involved in the calcium signalling and arachidonic acid metabolism pathways, respectively. The gene* BDKRB1* is a receptor that specifically binds bradykinin, which is a nine-amino acid peptide generated under pathophysiological conditions, such as inflammation [40]; its activation is involved in producing the proinflammatory cytokines IL-1*β* and IL-6 [41, 42], neutrophil migration, and activation of various cell pathways [43]. Studies have shown* BDKRB1* activation and/or increased expression in several pathological states, including infection [44], allergy [45], arthritis [46], cancer [47], chronic pain and inflammation [42, 48], diabetes mellitus [49], and neurological disorders, such as epilepsy, stroke, multiple sclerosis [50], and Alzheimer's disease [51]. In Alzheimer's disease, which appears early in DS individuals [52],* BDKRB1* activation likely contributes to neuroinflammation [53].

Individuals with DS exhibit a higher infection frequency, especially in the upper respiratory tract; autoimmune diseases, such as hypothyroidism, celiac disease, and diabetes mellitus; premature neuroinflammatory processes; and an increased mortality rate related to sepsis [54]. Therefore, we expected increased* BDKRB1* expression in children with DS. However, the exclusion criteria were the absence of clinical signs of acute infection and absence of chronic infection. Thus, we suggest that individuals with DS may exhibit a lower basal expression level than individuals without the syndrome. In addition, for infections, even with an increase in the BDKRB1 gene expression, the cytokine production pathways, neutrophil migration, and activation levels for several cell-signalling pathways might be compromised. Furthermore, the mechanisms involved in the infection/inflammation processes may be less efficient, which may contribute to the clinical manifestation of the syndrome.

The gene* LTA4H* encodes a bifunctional zinc metalloprotein that converts leukotriene A4 (LTA4) into leukotriene B4 (LTB4) [55]. Leukotriene * *A4 also can * *yield leukotriene * *C4* * (LTC4)*   *by* * conjugation* * with* * reduced glutathione * *through the enzyme leukotriene * *C4* * (LTC4)* * synthase. LTC4 is converted* * to * *leukotriene* * D4* * (LTD4) that by sequential * *amino* *acid* * hydrolysis is converted into leukotriene* * E4 * *(LTE4) [56]. The leukotrienes (LTs) are powerful eicosanoid lipid mediators involved in acute and chronic inflammatory diseases, such as inflammatory bowel disease [57], chronic pulmonary neutrophilic inflammation [58], arthritis [59] asthma [60], and atherosclerosis [61].

Leukotrienes are the major products of arachidonic acid metabolism and are produced by inflammatory cells, including polymorphonuclear leukocytes, macrophages, and mast cells [56]. In particular, LTB4 is a powerful proinflammatory lipid mediator synthesized by immune cells and stimulates the production of several cytokines implicated in chronic inflammatory diseases and may play a role in recruiting inflammatory cells to the tissue lesion site [56]. Furthermore, LTB4 levels increases were associated with risk for pulmonary complications in multiply traumatized patients [62]. Thus, the altered* LTA4H* expression observed in this study suggests that individuals with DS may produce leukotrienes less efficiently, which impair the inflammatory response.

The CAM pathway is also worth discussing, which involves the genes* CD40LG*,* ITGB1*,* ITGAM*,* VCAM1*, and* ICAM1*. In this study, the gene* CD40LG* was expressed at lower levels in children with DS, confirming literature data such as the observations by Letourneau et al. [63] and Zampieri et al. [23] in fetal fibroblasts from monozygotic twins and in monocytes from peripheral blood taken from children with DS, respectively. The protein encoded by the* CD40LG* gene is a ligand for the CD40 receptor and is expressed on the surface of T-cells and regulates B-cell function. The interaction between CD40 and its ligand activates a proinflammatory signalling pathway [64]. Changes in the CD40/CD40LG signalling pathways lead to a type of congenital immunodeficiency characterized by low/absent IgG and IgA, normal circulating B lymphocytes, and increased IgM [64].

The genes* ITGB1* and* ITGAM* also exhibited lower expression levels in children with DS in our study. The* ITGB1* gene encodes a membrane receptor involved in cellular adhesion [65], and changes in its expression may impact the pathway that regulates cytoskeletal actin, which is involved in cell growth, survival, and motility [66]. Indeed, previous studies have shown that the number of total lymphocytes is lower in children with DS than in children without the syndrome [20]; in addition, lymphocyte maturation is impaired in these individuals [19]. The* ITGAM* gene encodes the integrin alpha-M chain; this domain contains integrin alpha and combines with the beta 2 chain (ITGB2) to form an integrin specific to leukocytes referred to as macrophage-1 receptor (Mac-1) leukocytes. The alpha-M of integrin beta 2 is important for neutrophil and monocyte adhesion to the stimulated endothelium and for phagocytosis of complement-coated particles [67].

The genes* VCAM1* and* ICAM1* showed increased expression in children with DS in this study and are closely related both structurally and functionally; they also encode cell-surface glycoproteins that are activated by cytokines in endothelial cells. These glycoproteins are ligands for integrins that participate in leukocyte adhesion to the endothelium [68, 69] and play an important role in signal transduction, especially proinflammatory pathways, through promoting the recruitment of inflammatory immune cells, such as macrophages and granulocytes [70]. The signal transduction mechanisms involved in ligation with antigen receptors in B and T-cells require a set of highly coordinated interactions that involve transmembrane and cytosolic proteins. Studies suggest that T-cell signalling changes in response to mitogens may produce abnormal lymphocyte proliferation in individuals with DS [71].

The* ICAM1* gene binds CD11 integrins, which are known as lymphocyte function-associated antigen 1 (LFA-1) [69], a receptor found on leukocytes [72]. When activated by the ICAM-1/LFA-1 pathway, leukocytes bind to endothelial cells and migrate through the tissues, which elicits an inflammatory response [73]. Due to its proinflammatory role, the ICAM1 protein has been observed at low concentrations across the membrane of leukocytes and endothelial cells under normal conditions [70].

Lin et al. [74] used a cell adhesion assay with recombinant ICAM-1 and observed increased LFA-1 expression in the lymphocytes of patients with DS as well as lower adhesion of these cells compared with patients without the syndrome. Changes in LFA-1 function may compromise lymphocyte activation and maturation [70]. These results suggest that more generalized pathological processes, such as premature senescence of the immune system or inefficient lymphocyte activation, and subsequent integrin dysfunction may underlie the immunological deficiencies observed in patients with DS.

In conclusion, in our study, we show that children with DS exhibit differential expression of genes related to inflammation that are not located on chromosome 21 compared with children without the syndrome. Altered expression of these genes, especially* BDKRB1* and* LTA4H*, may differentiate the case and control groups based on the inflammatory response, which plays an important role in DS pathogenesis.

## Figures and Tables

**Table 1:** Graphical representation of the 96-plex gene card containing 92 genes related to inflammation and four reference genes.

	1	2	3	4	5	6	7	8	9	10	11	12
A	18S^a^	GAPDH^a^	HPRT1^a^	GUSB^a^	A2M	ADRB1	ADRB2	ALOX12	ALOX5	ANXA1	ANXA3	ANXA5
B	KLK3	BDKRB1	BDKRB2	CACNA1C	CACNA1D	CACNA2D1	CACNB2	CACNB4	CASP1	CD40	CD40LG	CES1
C	LTB4R	MAPK14	NR3C1	HPGD	HRH1	HRH2	HTR3A	ICAM1	IL1R1	IL2RA	IL2RB	IL2RG
D	IL13	ITGAL	ITGAM	ITGB1	ITGB2	KLK1	KLK2	KLKB1	KNG1	LTA4H	LTC4S	MC2R
E	NFKB1	NOS2	PDE4A	PDE4B	PDE4C	PDE4D	PLA2G1B	PLA2G2A	PLA2G5	PLCB2	PLCB3	PLCB4
F	PLCD1	PLCG1	PLCG2	MAPK1	MAPK3	MAPK8	PTAFR	PTGDR	PTGER2	PTGER3	PTGFR	PTGIR
G	PTGIS	PTGS1	PTGS2	TBXA2R	TBXAS1	TNF	TNFRSF1A	TNFRSF1B	VCAM1	IL1R2	PLA2G7	PLA2G10
H	PLA2G4C	IL1RL1	HTR3B	TNFSF13B	CYSLTR1	HRH3	PLA2G2D	IL1RAPL2	KLK14	PLCE1	KLK15	LTB4R2

^a^Reference genes.

**Table 2:** Significantly different gene expression between children with and without DS.

Gene	Gene ID^a^	Gene name	Mean RQ	RQ log⁡2	*p* value
*BDKRB1*	623	*Bradykinin receptor B1*	0.0013	−9.6225	0.0003
*LTA4H*	4048	*Leukotriene A4 hydrolase*	0.0079	−6.9899	0.0005
*ITGAM*	3684	*Integrin, alpha-M (complement component 3 receptor 3 subunit)*	0.0364	−4.7811	0.0144
*ADRB2*	154	*Adrenoceptor beta 2, surface*	0.0543	−4.2037	0.0089
*CD40LG*	959	*CD40 ligand*	0.0737	−3.7613	0.0151
*ITGB1*	3688	*Integrin, beta 1*	0.0779	−3.6822	0.0399
*TNFRSF1B*	7133	*Tumor necrosis factor receptor superfamily, member 1B*	0.0829	−3.5917	0.0258
*TBXAS1*	6916	*Thromboxane A synthase 1 (platelet)*	0.1032	−3.2768	0.0428
*PLCB4*	5332	*Phospholipase C, beta 4*	8.7676	3.1322	0.0443
*ICAM1*	3383	*Intercellular adhesion molecule 1*	9.7116	3.2797	0.0268
*HTR3A*	3359	*5-Hydroxytryptamine (serotonin) receptor 3A, ionotropic*	9.8319	3.2975	0.0343
*ALOX12*	239	*Arachidonate 12-lipoxygenase*	10.6198	3.4087	0.0171
*PLA2G5*	5322	*Phospholipase A2, group V*	12.5688	3.6518	0.0423
*PDE4C*	5143	*Phosphodiesterase 4C, cAMP-specific*	19.2927	4.2700	0.0390
*PLCD1*	5333	*Phospholipase C, delta 1*	19.9510	4.3184	0.0449
*PLA2G2D*	26279	*Phospholipase A2, group IID*	21.3914	4.4190	0.0067
*ADRB1*	153	*Adrenoceptor beta 1*	24.2235	4.5983	0.0491
*CACNA1D*	776	*Calcium channel, voltage-dependent, L type, alpha 1D subunit*	25.3055	4.6614	0.0125
*CASP1*	834	*Caspase 1, apoptosis-related cysteine peptidase*	47.1421	5.5589	0.0479
*VCAM1*	7412	*Vascular cell adhesion molecule 1*	52.8885	5.7249	0.0243

^a^Gene Identification second-base gene data, the National Center for Biotechnology Information (NCBI, http://www.ncbi.nlm.nih.gov/gene).

**Table 3:** Metabolic pathways associated with the target genes (*p* < 0.05) in children with DS based on the Kyoto Encyclopedia of Genes and Genomes (KEGG) database.

Metabolic pathways	Genes	^a^ *p* value
Calcium signalling pathways	*ADRB2, ADRB1, PLCB4, PLCD1, BDKRB1, *and* CACNA1D*	0.0075
Cell adhesion molecules (CAMs)	*CD40LG, VCAM1, ICAM1, ITGB1, *and* ITGAM*	0.0179
Arachidonic acid metabolism	*TBXAS1, LTA4H, PLA2G2D, PLA2G5, *and* ALOX12*	0.0019

^a^Corrected by the method *Benjamini-Hochberg False Discovery Rate*.
